# Seronegative Autoimmune Encephalitis With Rapidly Progressive Parkinsonism

**DOI:** 10.7759/cureus.76114

**Published:** 2024-12-21

**Authors:** Richa Mallick, Abhijith Rao, Vasu Digra, Pramod Kumar, Avinash Chakrawarty

**Affiliations:** 1 Geriatric Medicine, All India Institute of Medical Sciences, New Delhi, New Delhi, IND

**Keywords:** neuroimaging, older adults, parkinsonism, rapidly progressive, seronegative autoimmune encephalitis

## Abstract

Seronegative autoimmune encephalitis is an infrequent entity in older patients, and its presentation as rapidly progressive parkinsonism is rarely seen in cases of autoimmune encephalitis. We present a case report of an older patient who presented with worsening Parkinson’s-like symptoms over a month. We highlight the importance of considering autoimmune encephalitis as a potential cause of rapidly progressive parkinsonism and the importance of radiological findings when the entire autoimmune panel is negative in such patients.

## Introduction

Autoimmune encephalitis is a central nervous system disorder (CNS) caused by antibodies that attack the brain’s nerve cells [1]. These antibodies can damage the cells and cause inflammation, leading to various symptoms, including seizures, psychosis, confusion, changes in personality, and intellectual decline. Autoimmune encephalitis-related antibodies can be divided into two categories: antibodies against neuronal surface receptors (anti-NMDAR, anti-GABA-B receptor, anti-AMPAR, and anti-LGI1) and antibodies against neuronal intracellular antigens (anti-Hu, anti-Yo, anti-Ri, anti-Ma2, and anti-CV2) [2]. Parkinsonism in autoimmune encephalitis is an infrequent clinical presentation and is usually associated with the presence of antibodies or malignancy [3]. We report the case of an older patient who presented with rapidly worsening Parkinson’s-like symptoms and was diagnosed with seronegative autoimmune encephalitis without any malignancy.

## Case presentation

A 73-year-old male patient with a history of hypertension, hypothyroidism, and coronary artery disease presented with one month of fatigue, low mood, decreased oral intake, and reduced self-care and hygiene. The patient's relatives noticed tremors in both his upper limbs for ten days. These tremors later spread to both his legs. The patient also experienced increased stiffness in all four limbs, slow, short steps, slowing of movement, and abnormal orofacial movements for seven days. Three days before admission, he developed a change in behavior, irrelevant speech, forgetfulness, and reduced sleep.

On examination, he was conscious, not oriented to time, place, and person, and afebrile. His vitals were stable. Nervous system examination revealed bilateral coarse resting tremors with bradykinesia and cogwheel rigidity in bilateral upper and lower limbs with flexor plantar. A finger-nose test revealed bilateral past pointing with tremors. Gait evaluation revealed a wide-based gait, with swaying towards both sides. The rest of the examination was normal.

His initial investigations revealed hyponatremia (124 mg/dl) with the syndrome of inappropriate antidiuretic hormone secretion (SIADH), which was corrected. Complete blood count, renal and liver function tests and creatine phosphokinase levels were within normal limits. Mini-Mental State Examination was 13/30. Two days after hospitalization, the patient developed dysarthria, and rigidity increased. Because of worsening Parkinsonism, a levodopa trial was given, but there was no improvement.

Magnetic resonance imaging (MRI) of the brain revealed age-related cerebral atrophy. Cerebrospinal fluid examination revealed TLC-1 (100% lymphocyte), slightly elevated protein (75 mg/dL), and a normal sugar of 79 mg/dL (blood RBS: 136 mg/dL). Tests for viral encephalitis, bacterial, fungal, tuberculosis, syphilis, Lyme disease, and cytology were negative. ADA was 1.4. Serum autoimmune and paraneoplastic panels were negative (Table 1).

**Table 1:** Serum autoimmune and paraneoplastic profile tested

Autoantibody	Result
Anti-Hu (ANNA-1)	Negative
Anti-Ri (ANNA-2)	Negative
Anti-Yo (PCA-1)	Negative
Anti-CV-2 (Anti CRMP5)	Negative
Anti-PNMA2 (Ma2/Ta)	Negative
Anti-amphiphysin	Negative
Anti-SOX1 (AGNA)	Negative
Anti-Tr (PCA-Tr)	Negative
Anti-GAD65	Negative
Anti Zic4	Negative
Anti-titin	Negative
Anti-Recoverin	Negative
NMDA Ab	Negative
AMPA1 Ab	Negative
AMPA2 Ab	Negative
CASPR Ab	Negative
LGL-1 Ab	Negative
GABA-B receptor Ab	Negative

**Figure 1:**
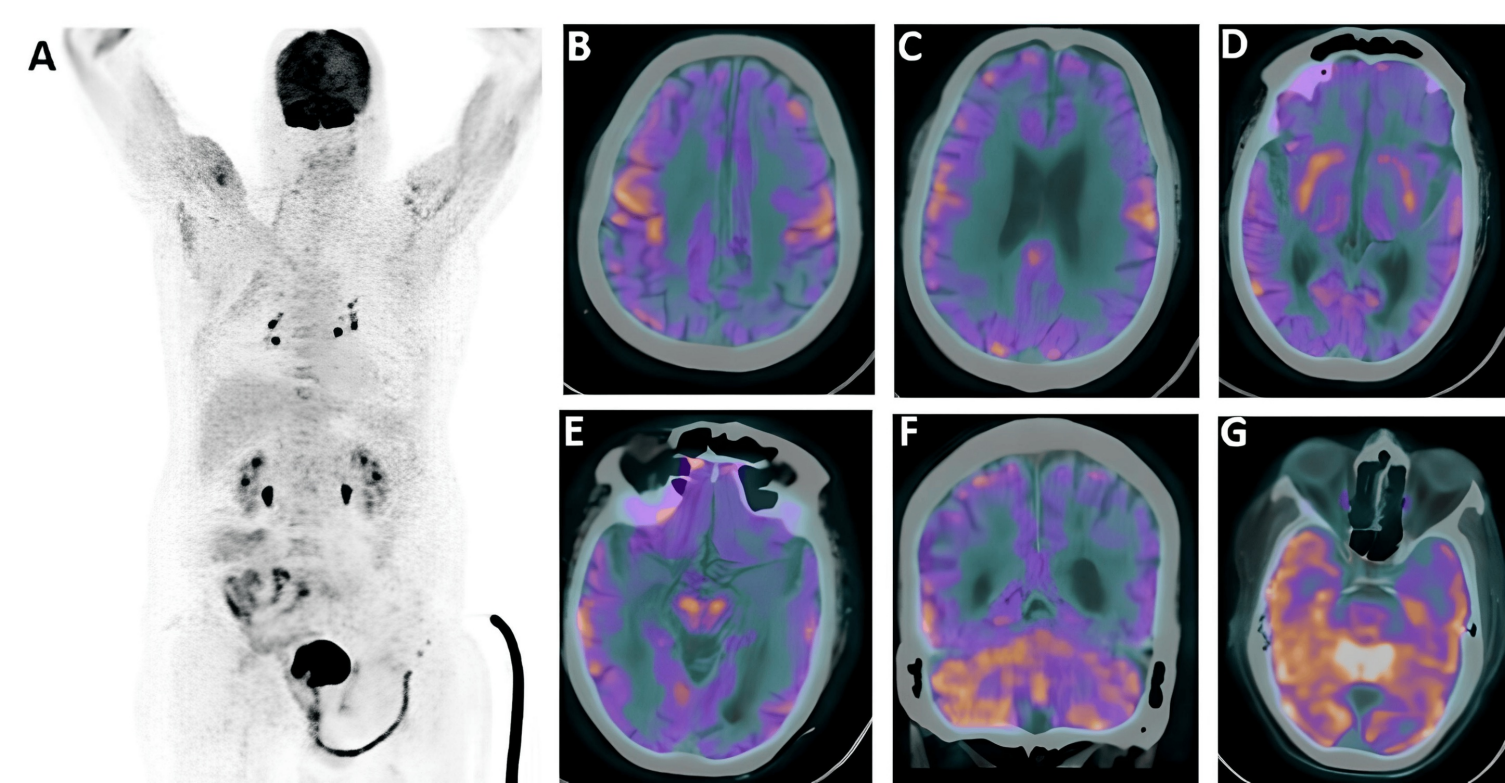
Maximum intensity projection (MIP) image of 18F-FDG PET/CT (A) showing physiological tracer uptake with no abnormal uptake. Fused PET/CT images show hypometabolism in bilateral frontal (B), bilateral parietal (C,D), and lateral temporal lobes (E) with hypermetabolism in the midbrain (E), bilateral cerebellum (F), and bilateral mesial temporal lobes (G). These findings are suspicious of autoimmune encephalitis.

Serum IgLon5 antibody also came back negative. A positron emission tomography (PET) scan of the brain revealed hypometabolism in bilateral frontal, parietal, temporal, and occipital cortices, suggesting autoimmune etiology (Figure 1).

Since the patient was rapidly deteriorating, it was decided to treat based on the PET scan, and the patient was started on pulse steroid therapy. After five days, there was an improvement in his symptoms. The patient was shifted to an oral tapering dose of steroids. Physical rehabilitation was initiated to improve his functional status. At discharge, the patient could perform his basic activities of daily living independently. At the six-month follow-up, the patient has tapered off steroids, maintaining all his activities of daily living independently.

## Discussion

This case report describes an older adult who presented with rapidly progressive Parkinsonism and was diagnosed with autoimmune encephalitis. Autoimmune encephalitis is a rare condition that is characterized by inflammation of the brain due to an autoimmune reaction. The differential diagnosis for rapidly progressive Parkinsonism is broad and includes neurodegenerative disorders (e.g., idiopathic Parkinson's disease, multiple system atrophy), vascular and drug-induced Parkinsonism, Wilson's disease, metabolic disorders (e.g., mitochondrial disorders, lysosomal storage diseases), infectious causes (e.g., viral encephalitis, COVID-19), infections (encephalitis, HIV, syphilis, Lyme’s disease), and autoimmune encephalitis [4-7]. Considering the rarity of autoimmune encephalitis, it is often overlooked or misdiagnosed, leading to delays in appropriate management.

Autoimmune encephalitis is a relatively rare condition, but its recognition and diagnosis are crucial due to the potential for rapid progression and significant morbidity [1]. The exact mechanism by which autoimmune encephalitis causes Parkinsonism is not fully understood. However, it is thought that the inflammation in the brain damages the neurons that produce dopamine, a neurotransmitter essential for movement [8]. This damage can lead to the symptoms of Parkinsonism, such as tremors, rigidity, and bradykinesia. Seronegative autoimmune encephalitis refers to a condition in which there is no detectable presence of autoantibodies despite exhibiting clinical symptoms consistent with autoimmune encephalitis. Diagnostic criteria for antibody-negative but probable Autoimmune encephalitis is rapid progression (less than 3 months) of working memory deficits (short-term memory loss), altered mental status, or psychiatric symptoms; exclusion of well-defined syndromes of autoimmune encephalitis; absence of well-characterized autoantibodies in serum and CSF; and at least two of the following criteria: MRI abnormalities suggestive of autoimmune encephalitis, CSF pleocytosis, CSF-specific oligoclonal bands or elevated CSF IgG index, or both, brain biopsy showing inflammatory infiltrates and excluding other disorders (e.g., tumor), and reasonable exclusion of alternative causes [9].

In our specific case, the importance of imaging in diagnosing seronegative autoimmune encephalitis becomes apparent, which is supported by prior literature [10,11].

## Conclusions

This case report highlights the importance of considering autoimmune encephalitis as a potential cause of rapidly progressive Parkinsonism in older adults. While autoimmune encephalitis is rare, its early identification is essential for timely intervention and appropriate management. Geriatricians and neurologists should maintain a high index of suspicion for autoimmune encephalitis in patients presenting with rapidly progressive Parkinson’s-like symptoms. Prompt recognition and diagnosis of autoimmune encephalitis can facilitate targeted immunotherapy and improve patient outcomes, emphasizing the significance of this case report for clinicians in these specialties.
